# Reply to Zhang et al.

**DOI:** 10.1111/pai.70133

**Published:** 2025-06-30

**Authors:** Raffi Tachdjian, Aleena Banerji, Paula J. Busse, Nancy Agmon‐Levin, John Anderson, Mauro Cancian, Giuseppe Spadaro, Carmen Enciu, Daniel Nova Estepan, Natalie Khutoryansky, Siddharth Jain, Andreas Recke

**Affiliations:** ^1^ Division of Allergy, Immunology and Rheumatology UCLA School of Medicine Los Angeles California USA; ^2^ Providence St. John Medical Center Santa Monica California USA; ^3^ Division of Rheumatology, Allergy and Immunology, Department of Medicine, Massachusetts General Hospital Harvard Medical School Boston Massachusetts USA; ^4^ Division of Allergy and Clinical Immunology Icahn School of Medicine at Mount Sinai New York New York USA; ^5^ Clinical Immunology, Angioedema and Allergy Institute, The Center for Autoimmune Diseases Sheba Medical Center, Tel HaShomer Hospital Ramat‐Gan Israel; ^6^ AllerVie Health Birmingham Alabama USA; ^7^ Department of Systems Medicine University Hospital of Padua Padua Italy; ^8^ Department of Translational Medical Sciences University of Naples Federico II Naples Italy; ^9^ Center for Basic and Clinical Immunology Research (CISI), WAO Center of Excellence University of Naples Federico II Naples Italy; ^10^ Takeda Development Center Americas, Inc. Lexington Massachusetts USA; ^11^ Department of Dermatology, Allergology and Venereology University of Lübeck Lübeck Germany

To the Editor,

We thank Zhang et al.^1^ for their comments on our article “Effective long‐term prophylaxis with lanadelumab in adolescents with hereditary angioedema: EMPOWER/ENABLE”.^2^ In their correspondence, Zhang et al. correctly noted that sex hormone fluctuations may affect the natural history of hereditary angioedema (HAE) during adolescence and suggest that an extended observation period beyond 36 months would clarify the durability of lanadelumab during any disease instability tied to hormonal changes. Unfortunately, the EMPOWER and ENABLE studies were not designed for follow‐up longer than 3 years, and both studies have now concluded. Additionally, Zhang et al. suggested that lanadelumab, as a monoclonal antibody, may have what they describe as a delayed immunogenic effect, and, as such, long‐term safety monitoring is needed. Although immunogenicity has been identified as a potential risk associated with therapeutic monoclonal antibodies in general, the potential immunogenic risk with lanadelumab has been demonstrated to be low for patients with HAE, including adolescents. In the long‐term HELP open‐label extension study, which included adolescent patients, the occurrence of anti‐drug antibodies and neutralizing antibodies were infrequent and had no impact on the efficacy or safety of lanadelumab.^3^ Additionally, continued monitoring assessed via periodic benefit–risk evaluation reports and periodic safety update reports are required for lanadelumab as part of pharmacovigilance obligations as for any product approved by regulatory authorities. To date, the safety profile of lanadelumab remains consistent with that established during clinical trials and there is no safety signal pertaining to the potential immunogenic risk with lanadelumab that impacts efficacy or safety. We agree that longer follow‐up could provide more data for this important population whose characteristics, such as hormones, growth, and weight, are changing while experiencing additional stressors, such as important education examinations, and encourage the wider community to participate in existing and new registries where such data can be collected and reported to help advance the understanding of the disease.

Zhang et al. also emphasized that data on quality of life (QoL) should be considered for a holistic assessment of lanadelumab's clinical value in adolescents. The health‐related (HR) QoL data collected in EMPOWER and ENABLE add important information to the body of literature on lanadelumab in adolescents with HAE. To date, only data from the ENABLE Study have been analyzed post hoc from the adolescent subpopulation. HRQoL was assessed using the Angioedema QoL (AE‐QoL) questionnaire, administered at baseline, every month for 3 months, and then every 6 months thereafter. At baseline, the median total AE‐QoL score was 39.7 among adolescents who completed the questionnaire, indicating moderate to large HRQoL impairment.^4^ The median AE‐QoL total score decreased by >10 points at Month 1, where a change of 6 points is considered the minimal clinically important difference,^4^ and improvements were sustained thereafter in patients with data at the relevant timepoints. In detail, median (interquartile range [IQR]) AE‐QoL scores improved from 39.7 (17.6–70.6) at baseline (*n* = 7) to 27.9 (1.5–61.8) at Month 1 (*n* = 6), 17.6 (4.4–32.4) at Month 12 (*n* = 5), and 17.6 (11.8–35.3) at Month 36 (*n* = 3). Additionally, the Angioedema Control Test (AECT) questionnaire, evaluating patient perception of disease control, showed that all adolescents completing the questionnaire had poorly controlled disease at baseline, with an AECT total score <10 (median, 5.5). The AECT total score increased to 16.0 at Month 2 (*n* = 5), representing well‐controlled disease, where a minimal clinically important difference is a 3‐point change.^5^ Patient perception of well‐controlled disease was maintained thereafter: specifically, median (IQR) AECT total scores were 5.5 (3.0–8.0) at baseline (*n* = 6), 16.0 (10.0–16.0) at Month 2 (*n* = 5), and 12.0 (12.0–16.0) at Month 36 (*n* = 3). These data highlight the benefits of lanadelumab, in addition to attack‐rate reduction, in adolescents with HAE and reinforce its use as a first‐line long‐term prophylactic treatment, as recommended by the WAO/EAACI guidelines.^6^ Although international guidelines suggest all patients receiving long‐term prophylaxis be routinely monitored to assess the impact of HAE, in addition to the disease activity and its control, to inform optimization of treatment dosages and outcomes, there is an opportunity to better emphasize and encourage regular implementation of patient‐reported tools in clinical practice.

Zhang et al. suggested two statistical approaches as a way to add value to the reporting of our findings. Real‐world observational studies, by their nature, lack in‐study comparator groups. Propensity score matching is a useful approach in datasets with a large sample size but could reduce the size of our already somewhat limited subpopulation due to unmatched individuals. Zhang et al. also suggested including confounding variables in a multivariate regression model, specifically sex hormone levels, pubertal stage, and prior prophylaxis use. Owing to the noninterventional nature of the EMPOWER and ENABLE Studies, sex hormone levels were not measured and Tanner stage was not assessed. Inclusion of Tanner staging may limit willingness of adolescents to enroll into studies, which can be especially problematic in studies with rare diseases, where it is important to minimize barriers to enrollment. Prior prophylaxis use was captured in ENABLE and EMPOWER but not incorporated into a statistical model because data from other studies, such as HELP, showed no difference between outcomes among patients with or without prior long‐term prophylaxis use.^7^ Subdividing an already small population to calculate attack rates according to prior prophylaxis use would likely have limited interpretability.

We thank Zhang et al. for their thoughtful comments, constructive critique, and conclusion that our analysis provided convincing evidence of lanadelumab's effectiveness and safety among adolescents with HAE. We also appreciate the opportunity to add more data to the limited literature on adolescents with HAE, which can support future research.

## AUTHOR CONTRIBUTIONS

**Raffi Tachdjian:** Writing – original draft; writing – review and editing. **Aleena Banerji:** Writing – original draft; writing – review and editing. **Paula J. Busse:** Writing – original draft; writing – review and editing. **Nancy Agmon‐Levin:** Writing – original draft; writing – review and editing. **John Anderson:** Writing – original draft; writing – review and editing. **Mauro Cancian:** Writing – original draft; writing – review and editing. **Giuseppe Spadaro:** Writing – original draft; writing – review and editing. **Carmen Enciu:** Writing – original draft; writing – review and editing. **Daniel Nova Estepan:** Writing – original draft; writing – review and editing. **Natalie Khutoryansky:** Writing – original draft; writing – review and editing. **Siddharth Jain:** Writing – original draft; writing – review and editing. **Andreas Recke:** Writing – original draft; writing – review and editing.

## FUNDING INFORMATION

The studies and this response were sponsored/funded by Takeda Development Center Americas, Inc., Lexington, MA, USA.

## DISCLOSURES

RT has been a clinical investigator/consultant for Astria Therapeutics, BioCryst, CSL Behring, Ionis Pharmaceuticals, KalVista Pharmaceuticals, Pharming, Pharvaris, and Takeda; a speaker for BioCryst, CSL Behring, Pharming, and Takeda; and has served on advisory boards for BioCryst, CSL Behring, Ionis Pharmaceuticals, KalVista Pharmaceuticals, Pharming, and Takeda. AB has received institutional research/study support from Astria Therapeutics, Ionis Pharmaceuticals, and Takeda; and/or honoraria for consulting from ADARx, Astria Therapeutics, BioCryst, CSL Behring, Intellia Therapeutics, KalVista Pharmaceuticals, and Takeda. PJB has received research support and served on advisory boards for BioCryst, CSL Behring, and Takeda; and is a consultant for CVS Pharmacy, Medscape, Novartis, and Regeneron. NA‐L has no conflicts of interest to disclose. JA is a speaker bureau member for BioCryst, CSL Behring, Pharming, and Takeda; has received consulting fees from and is a clinical trial investigator for BioCryst, BioMarin, CSL Behring, KalVista Pharmaceuticals, Pharming, Pharvaris, and Takeda; and has received consulting fees from Cycle Pharma. MC reports personal fees from BioCryst, CSL Behring, KalVista Pharmaceuticals, Pharvaris, and Takeda. GS has received speaker/consultancy fees from AstraZeneca, Chiesi Farmaceutici, CSL Behring, GSK, Sanofi, and Takeda. CE, DNE, NK, and SJ are employees of Takeda Development Center Americas, Inc. and hold stock/stock options in Takeda Pharmaceutical Company Limited. AR has received research grants from Deutsche Forschungsgemeinschaft and EUROIMMUN; travel and conference support from BioCryst, Novartis, Pharming, Stallergenes Greer, and Takeda; speaker honoraria from Bencard, BioCryst, CSL Behring, EUROIMMUN, Novartis, and Takeda; and served as a consultant or participated in advisory boards for BioCryst, CSL Behring, Novartis, Swedish Orphan Biovitrum, and Takeda.

## PEER REVIEW

The peer review history for this article is available at https://www.webofscience.com/api/gateway/wos/peer‐review/10.1111/pai.70133.
